# An investigation of the relation between online and offline violence: methodological and theoretical considerations

**DOI:** 10.3389/fpsyg.2025.1623246

**Published:** 2025-12-16

**Authors:** Melanie G. Molenaar, Frank M. Weerman, Ivy N. Defoe

**Affiliations:** 1 BMC, Amersfoort, Netherlands; 2 Netherlands Institute for the Study of Crime and Law Enforcement, Amsterdam, Netherlands; 3 Department of Law, Society and Crime, Erasmus University, Rotterdam, Netherlands; 4 Forensic Child and Youth Care Sciences, Faculty of Social and Behavior Sciences, Institute of Child Development and Education, University of Amsterdam, Amsterdam, Netherlands; 5 Interdisciplinary Research Team on Internet and Society, Faculty of Social Studies, Masaryk University, Jostova, Czechia

**Keywords:** social media, youth, violence, developmental stage, peer deviancy

## Abstract

**Introduction:**

Research focusing on whether exposure to “online” violence via social media (exposure to violent content, such as videos of beatings or fights) predicts the use of physical “offline” violence (from hitting and threatening someone to violence against objects) in real-life is scarce and consists of mostly qualitative studies. The current quantitative study was designed to investigate this association while controlling for gender, and used the General Aggression Model (GAM) and the Developmental Neuro-ecological Risk-taking Model (DNERM) for its theoretical framework. Additionally, to what extent developmental stage (adolescents vs. young adults) and (offline) peer deviancy moderate this association was explored. We further used two separate measures of exposure to violent social media: (1) violent social media exposure in general and (2) violent social media exposure by friends.

**Methods:**

A total of 292 ethnically- and socio-economically-diverse Dutch youth between 16 and 24 years (M = 19.52; SD = 3.218) participated in a survey study.

**Results:**

Consistent with our theoretical frameworks, stepwise regression analyses supported the main hypothesis, as a significant association was found between the two measures of exposure to violent social media content and youth violence in real-life. However, we only found partial support for the moderation hypotheses: while peer deviancy was not a significant moderator, it was a significant predictor. Additionally, developmental stage was a significant predictor, and it was a significant moderator—but only when the “violent social media exposure in general” measure was used. Specifically, in support of DNERM, the results revealed that the link between violent social media exposure in general was stronger for adolescents (versus emerging adults).

**Discussion:**

The current results demonstrate for the first time that when it comes to the effect of violent social media exposure on real-life violence, it may matter who (friends versus non-friends) is posting the violent content, but it also matters who the audience [e.g., adolescents versus (emerging) adults] is, and whether peer deviancy is already taken into account. Experimental and longitudinal research on this topic are necessary to further establish these findings. Methodological and theoretical considerations when investigating such online influences are discussed to guide future research on this topic.

## 1 Introduction

Social media use has grown rapidly in the last 20 years. For example, almost all Dutch youth (97%) between ages 12 and 24- are active on social media (CBS, 2020c). Social media platforms such as Snapchat, TikTok, Facebook, Instagram and WhatsApp can be used to share information with family, friends, and strangers. Positive examples of use include chatting with friends, uploading photos and music or applying for a job (CBS, 2020b). However, also negative types of content are shared. These include depictions and registrations of violence, such as stabbing incidents, humiliating beatings and illegal ‘hood fights’. For example, in the Netherlands the attack on a well-known Dutch crime reporter was an example of a violent event, that was recorded and posted on social media, accessible for the rest of the world. More recent examples include the violent acts after the Ajax-Maccabi football game in Amsterdam, which were recorded and posted on social media, accessible for the rest of the world (New York Times, 2024). Considering that Statistics Netherlands (CBS) reported that virtually all (99,8%) 15-24 years olds in 2024 used the internet in the past 12 months, there is a great chance that these youth are also exposed to such violent content (CBS, 2025).

Decades of research heavily discussed the potential effects of traditional media exposure to violence on aggression and own violence in real-life (Barker and Petley, 2002). Most studies find an effect on real-life violent behavior (Anderson et al., 2003, 2015), although the effects might be exaggerated in the past and are probably small (Ferguson and Kilburn, 2009; Savage and Yancey, 2008). There is also ample evidence for a modest effect of violent videogames on aggressive behavior and violence (Burkhardt and Lenhard, 2022; Gentile and Stone, 2005; Prescott et al., 2018). Much less research has explored the effects of social media exposure to violence on aggression and violent behavior. Available studies are often *qualitative*, and focus on the role of violent content on social media in the construction of street or gang identity and conflict escalation (see, e.g., Fernández-Planells et al., 2021; Patton et al., 2014; Roks and Van den Broek, 2020). *Quantitative* research on the effects of violent social media content is still sparce. Nevertheless, a cross-sectional survey study on online risky content found a significant association between coming across content encouraging violence and individual violent behavior, also when peer violence was included as a control variable (Branley and Covey, 2017). Also, a longitudinal survey study (McCuddy, 2021) on online effects on delinquency investigated the association between social media *support* for violence from online friends and individual physical violent behavior. After controlling for individual risk factors, including peer delinquency, there was no statistical significant independent effect in this study. However, changes in online support for violence were significantly and independently related to changes in individual violence. Of note is that the latter study only contained middle-school-aged children (typically between 11 and 13 years) (McCuddy, 2021), whereas the former only included (emerging) adults (Branley and Covey, 2017). It is crucial to replicate the findings of these studies in a more developmentally diverse sample to test for age differences for a more thorough understanding as both violence and the strength of social influence are age dependent (e.g., see Steinberg, 2007). Hence, to fill these gaps in the literature, the current study uses a sample that spans adolescence to emerging adulthood and considers developmental stage as a moderator in the potential relation between exposure to violent social media of others and youth violence in real-life.

### 1.1 (Social) Media effects on violence

In the literature, violence is defined and operationalized in diverging ways. Broadly speaking, there are two perspectives on violence (Bufacchi, 2005). Broad/comprehensive conceptualizations of violence includes multiple types of harms to others or themselves, or even symbolic violence or ‘reductions of human being’ (Stanko, 2003; Schinkel, 2010). Narrow/minimalist conceptions focus on concrete actions of physical force and injury towards other people (Coady, 1985; Geras, 1990). In this manuscript we use a relatively minimalist conception of violence and focus on physical injury, as well as threats or aggressive behavior suggesting the use of violence. We define violence as “any form of behavior by an individual that intentionally threatens to or does cause physical, sexual or psychological harm to others” (Stanko, 2001). Steger (2013, p. 13) argues that ‘violence is the intentional infliction of physical or psychological injury on a person or persons’. Violence is more severe than aggression, and the (risk of) injury is greater (Marcus, 2007).

According to the Cambridge Dictionary (n.d.) social media are “forms of media that allow people to communicate and share information using the internet or mobile phones.” A substantial difference between online and real-life is the possibility of editing the content before posting. This gives social media users the chance to portray themselves better than they really are (Ditchfield, 2020). Importantly, social media makes it easy to find deviant peers (groups) and plan risky behavior in real-life (Vannucci et al., 2020). Finally, uploading violent videos and live streaming conflicts are examples of violent activities on social media (Patton et al., 2014; Elsaesser et al., 2021). Social media are different from other media, since users are not only recipients but also generators of the content (Gezgin, 2014). Unfortunately, no empirical quantitative studies could be found linking social media violence exposure to real-life violence in youth. Therefore, only research addressing the relation between other forms of violent media (e.g., films, video games) and violence in real-life will be reviewed here.

In films and videogames, aggressive and/or violent characters are often rewarded and seen as heroes. For example, an experimental study focusing on a sample of 99 Dutch boys, found that boys want to be like their ‘heroes’ and thus model the behavior of these violent characters, seen in films or videogames. This identification made the boys in the study more violent in real-life (Konijn et al., 2007). Additionally, another experimental study (*N* = 101) compared violent versus neutral video games. A significant increase in aggressive behavior after playing violent video games was found (Hollingdale and Greitemeyer, 2014). Finally, a two-year longitudinal study showed that youth (*N* = 314) became more violent and/or showed more delinquent behavior after watching violent films and programs or playing violent games (Hopf et al., 2008). However, although numerous studies address the impact of violent media see, e.g., the overview of Anderson et al. (2015), the impact of specifically violence on social media remains understudied. The current study will investigate whether the established findings about the impact of traditional media exposure to violence can be generalized to exposure to violence on social media.

### 1.2 Conceptual and theoretical background

Much of the research up until now has used social learning theory (Bandura, 1977) as a general account for how exposure to violence can contribute to the development of violent behavior. However, of note is that social learning theory (Bandura, 1977) was not originally developed to explain online social influences, which may be different from how such influences unfold in real-life (see: Defoe, 2021). Nevertheless, according to the social learning theory Bandura (1977), people observe and model behavior of others and learn in a social context what is expected from them through punishment and rewards. The criminological version of this theory (Akers, 1973)—which is also not specifically on online influences—states that deviant others can provide the attitudes and techniques that are necessary for rule breaking, provide social reinforcements for this behavior and role models that can be imitated. This theory, (Akers, 1973) builds on Bandura’s psychological social learning approach but also on the differential association theory of Sutherland (1947). The differential association theory (Sutherland, 1947) is another criminological theory that postulates that deviant and delinquent behavior is learned through the transmission of motives, attitudes, rationalizations, and techniques, in interaction with others, particularly in an intimate group. Although all the above mentioned theories were not developed to describe the effects of online media influences, they can be used as a foundation to understand such influences.

An approach that *does* specifically hypothesize about media influences in relation to violence, is the General Aggression Model (GAM). Among many other features, GAM explains how exposure to violent media increases aggression and indirectly violence. The model is divided into three stages: Stage 1 contains the inputs; namely the risk and protective factors that characterize a person and/or situation (e.g., violent media). Stage 2 addresses the routes; how do the inputs influence an individual’s affect (e.g., hostile/angry), cognition (e.g., aggressive thoughts) and arousal. Stage 3 focuses on the outcomes; here the person decides whether to respond in an aggressive or nonaggressive way (see Anderson and Bushman, 2002; Allen et al., 2018; DeWall et al., 2011). Extrapolating from this model, watching violent media can increase aggression and violence in various ways (e.g., increasing aggressive thoughts, getting used to violence) (Anderson and Bushman, 2018). Of note however, is that GAM was not developed to explain *social* media influences specifically—which as noted above—is a unique media type, and it is even distinct from other online media types as it allows users to engage with the content they are being exposed to.

A more recent hybrid psychological-criminological model, the Developmental Neuro-Ecological Risk-taking Model (DNERM; Defoe, 2021), can also be used as a theoretical approach to understand the relation between specifically social media violent content of others and real-world behavior in another. DNERM distinguishes offline “real-life” versus online influences on individual risk behaviors. DNERM postulates that both direct exposure to risk behaviors in real-life and indirect exposure to risk behaviors via (social) media can be used as a training ground where risk behavior (e.g., antisocial behavior) is learnt (Defoe, 2021). Additionally, indirect exposure to a cue of violence on social media may elicit the desire, normalization and acceptance by youth to try out such behaviors in real-life (Defoe, 2021). The model specifically addresses how these social learning and cue-reactivity processes are involved in the development of maladaptive risk behavior (e.g., delinquency including violence) during the youth period (11–24 years old) (Defoe, 2021). It is further theorized by DNERM that effects of such “risk exposure” may be moderated by self-control of the observer. The self-control of an individual increases with age/development (Defoe, 2021) and adolescents are still developing their identity and are curious about the (online) world around them (Defoe, 2021). Thus, it can be extrapolated from DNERM that youth would engage in more violence than older youth, and that (online) social influences would also be stronger for younger youth versus older youth. Extrapolating from DNERM, the current study considers a potential moderating role of developmental stage in the association between exposure to violent social media and violence in real-life. Two developmental stages are distinguished, namely adolescents (16–17-year-olds) and emerging adults (18–24-years-old) (see, e.g., Arnett et al., 2014).

During adolescence youth begin to distance themselves from their parents, while peers become more important for influencing youth’s behavior (see, e.g., Warr, 2002; Soh et al., 2018). In the process of adolescents distancing themselves from their parents, they start to make their own decisions independent of their parents’ guidance. However, because of their inexperience, support is often needed. This support can be found in peer groups, where peers share their beliefs and interests and where they are able to form their own identity, and a positive self-concept (Sussman et al., 2007). Peer risk behavior such as peer violence is a well-established predictor of adolescents’ own risk behavior (Albert and Steinberg, 2011). Research has shown for a long time that having violent/delinquent peers is a strong correlate of individual violence and delinquency (see, e.g., Warr, 2002; McGloin and Thomas, 2019).

It is important to note that a correlation does not necessarily mean that having deviant or violent peers indeed enhance violence among individuals. In the criminology and broader sociology literature on peers and deviant behavior, the causality of the relation has been heavily debated. According to some scholars, deviant peers are key to understanding the development of deviancy (Akers, 2009; Warr, 2002). Particularly during adolescence, processes of social learning and group processes may induce deviant and violent behavior situationally and over time. Other scholars have argued that the relation between deviant peers and own deviancy is largely spurious (Hirschi, 1969; Kandel, 1978). Adolescents who already have a tendency towards deviance and violence may have a strong preference to select friends who are similar to them. Based on research, it seems that both processes, influence and selection, play a role in the correlation between deviant peers and individual behavior (Gallupe et al., 2019).

Provided that there is an effect of online exposure to violence on individual behavior, it is possible that youth with deviant or violent peers will be the ones who will be affected more by violent social media exposure. In at least one study (*N* = 499), peer violence was considered as a moderating factor in the relation between violent traditional media use (e.g., violent television programs or videogames) and aggressive behavior (e.g., fighting). Youth with violent peers showed a higher risk of developing aggressive behavior after watching violent media than youth without these violent peers (Fikkers, 2016). A distinction must be made between the exposure to “traditional” media in the before mentioned study and social media exposure in the current study. For example, watching (violent) television programs is a passive action, while playing (violent) videogames is more interactive, and thus making it more controversial as it may be considered to have a more *direct* influence component on behavior. The possibility to not only interact but modify and generate new (violent) content on top of the social aspect of social media brings even another different dimension into the equation (Gezgin, 2014; Defoe, 2021). And hence DNERM posits that social media influences (versus other media influences) would be most influential for real-world behavior (Defoe, 2021). Taken together, similar to well-documented influences of other forms of media, we expect real-world effects for social media exposure. Furthermore, we also explore whether the association between social media violence and violence in real-life will be stronger for youth with deviant peers in real-life. This hypothesis can be extrapolated from DNERM as well, since this framework suggests that “offline” social influences (e.g., peer deviance observed in the “real world”) would be more dominant than (online) media influences on real-world behavior (Defoe, 2021).

### 1.3 Present study

Building on especially DNERM and GAM, the current study investigates the association between exposure to violent social media content and involvement in youth violence in real-life. Two types of social media exposure to violence are distinguished: exposure in general (model 1 & 3) and exposure coming from friends (model 2 & 4). We explore whether these types of exposure have differential effects on violence. The preliminary hypothesis (model 0) pertains to establishing whether youth who are exposed to more violent content on social media will have a higher risk of using violence in real-life. It does so by employing survey data from 292 Dutch youth between the ages of 16 and 24. Gender will function as a control variable, because much research has shown that boys are more involved in delinquent behavior than girls, particularly violence, and also that males are more often arrested for violent offenses than females (e.g., Esbensen et al., 2010). The second hypothesis (model 0) is that boys have a higher risk of using violence after exposure to social media violence than girls. Two variables that will be taken into consideration as controls and moderators are developmental stage and peer deviancy. It is well known that there is a relation between age and violent/delinquent behavior and that especially minor delinquent behavior peaks during the youth period (see, e.g., Farrington, 1986; Steinberg, 2007). Therefore, the third hypothesis (model 1 & 2) is that younger youth will have a higher risk of using more violent behavior after social media exposure than older youth. Further, many studies have shown that having deviant peers is strongly correlated with individual behavior (see, e.g., Warr, 2002; Steinberg, 2007). The final hypothesis (model 3 & 4) that is formulated is that youth with more deviant peers will have a higher risk of using violence after being exposed to social media violence than youth with fewer deviant peers.

## 2 Methods

### 2.1 Participants

This study focusses on the topic of violence, although the broader data-collection for this project covered additional risk behavior. A total of 292 Dutch participants between the ages of 16–24 (M = 19.52; SD = 3.218) participated in the study. For the analyses including developmental stage the sample was grouped as follows: 0 = adolescents aged 16–17 yrs. (44.3%) and 1 = emerging adults ages 18–24 yrs. (50.3%). The sample consisted of 184 females (69.7%) and 78 males (29.5%). A total of 71.1% of the fathers and 73.5% of the mothers were born in the Netherlands, and thus the sample can be considered quite ethnically diverse. Roughly 60% of the parents finished tertiary education, namely 58.7% of the fathers [15.8% secondary vocational education (MBO in Dutch), 25.2% applied university (HBO in Dutch) and 17.7% university (WO in Dutch)] and 57.3% of the mothers (25.9% secondary vocational education, 19.6% applied university and 11.8% university). These diverse educational levels reflect diversity in socioeconomic status within the Netherlands. Regarding educational level/tracks of the participants themselves, 124 participants were in secondary school, with the largest group (25.8%) following “preparatory middle-level applied education track” (“VMBO” in Dutch), 17% following a “higher general continued educational” track (“HAVO” in Dutch) and 4.2% of the participants following the “preparatory scientific education” track (“VWO” in Dutch). Additionally, a total of 140 participants were attending college/university (13.7% secondary vocational education; 14.8% applied university; 24.7% university). The remaining participants (*N* = 28) were currently not enrolled in school or college/university. Most of the participants lived with their parents or caregivers (69.7%). The rest lived in a student flat (6.4%), shared an apartment with friends or a roommate (6.8%), lived with their spouse (9.1%) or had their own place (8%).

Most of the participants used one or more social media platforms. The most popular was WhatsApp, namely 88.7% of the participants use this platform. Other popular platforms were Instagram (83.6%), Snapchat (79.1%), Facebook (43.8%) and Twitter (17.8%). When asked about everyday social media use, 57 participants (22.7%) used these social media 1–2 h per day, 137 participants (54.6%) 3–4 h per day and 57 participants (19.5%) used it 5 h a day or more. The most preferable type of content to view on social media was entertainment (56.7%), and other types were content posted by family or friends (22.4%), the news (9.4%), content from influencers (7.1%), or something else (4.3%) such as expanding the professional network.

### 2.2 Procedure

Approval from the ethics committee at the University of Amsterdam was granted for this study (id: 2022-CDE-14378). The questionnaire could be filled in anywhere, as long as the participant had access to a digital device and the internet. Via social media, and with the help of friends and family of the researchers, participants were recruited. To motivate youth to participate in the study, we included a voluntary prize raffle for a €20 voucher. To participate in this prize raffle, filling in an email address in the questionnaire was mandatory. To ensure anonymity, these email addresses were detached from the rest of the questionnaire. The email addresses were deleted 7 days after completing the data collection. Before starting with the questionnaire participants were asked to give informed consent.

### 2.3 Measures

#### 2.3.1 Youth Violence

We developed four questions for youth violence (the dependent variable) that tapped actual experience with physical violence in the last 12 months, such as videos of beatings or fights, threatening someone and violence against objects. To test if these items are appropriate to use to measure Youth Violence, a principal component analysis (PCA) with a varimax rotation was conducted (cf. Defoe et al., 2018). For Youth Violence, the Kaiser-Meyer-Olkon measure (KMO = 0.739) was good (Field, 2009) and the KMO values for the individual items were all above the acceptable limit of 0.5 (Field, 2009). The correlations between the items were large enough for a PCA (Field, 2009), since Bartlett’s test of sphericity *χ*^2^ (6) = 198.316, *p* < 0.001 was statistically significant. Only the first item met Kaiser’s criterion of >1. Item one had an initial eigenvalue of 2.259 and accounted for 56.487% of the total variance. All factor loadings represent substantive values (i.e., >0.40; Field, 2009). The factor loadings are shown in Table 1. The items had an adequate Cronbach’s Alpha of 0.732. An example item is: ‘How often did you use physical violence in the last 12 months, like hitting, kicking, stabbing or something else?’ Answers ranged from 0 = never, to 4 = more than 10 times. A mean score was computed for the analyses.

**Table 1:** Factor loadings for the youth violence scale.

Item	Factor loading
How often in the past 12 months have you done something that you knew was a legal violation (related to violence)?	0.764
How often in the past 12 months have you threatened someone with the intention of scaring them?	0.760
How often did you use physical violence in the past 12 months, like hitting, kicking, stabbing or something else?	0.744
How often in the past 12 months have you damaged or destroyed something that wasn’t yours, like a window, a lamppost, a bus or train or something else?	0.738

#### 2.3.2 Peer deviancy

We developed a three item scale for peer deviancy. Another PCA with a varimax rotation was conducted to support the use of this scale. The peer deviancy variable had an adequate KMO (KMO = 0.643) and again all the individual items had a KMO above the acceptable limit of 0.5 (Field, 2009). Bartlett’s test is statistically significant [*χ*^2^ (3) = 103.727, *p* < 0.001], thus using these items in a factor analysis is appropriate (Field, 2009). Item 1 met Kaiser’s criterion of >1. This item had an initial eigenvalue of 1.795 and accounted for 59.822% of the total variance. The factor loadings are shown in Table 2, all of them are >0.40, and thus substantive (Field, 2009). The scale had a Cronbach’s Alpha of 0.664, although lower than the generally minimum recommenced Alpha of 0.70, it is still within the acceptable limits (Field, 2018; Fraenkel and Wallen, 2006). The questions focused on peer deviancy, namely: 1. How many times in the last 12 months did one of your friends use physical violence, like hitting, kicking, stabbing or something else?; 2. How many times in the past 12 months did you help someone doing something illegal? The answers for these two questions ranged from 0 = never to 4 = more than 10 times. The final question was; 3. How often are you involved in a violent situation, like hitting, kicking, stabbing or something else? Answers for this question ranged from 0 = never to 3 = often. Since one of the questions has a different number of answer categories, *Z*-scores were used. With the standardized items, a mean score was calculated to conduct the analyses.

**Table 2:** Factor loadings for the peer deviancy scale.

Item	Factor loading
How many times in the past 12 months did one of your friends use physical violence, like hitting, kicking, stabbing or something else?	0.807
How many times in the past 12 months have you assisted someone else in committing something illegal?	0.799
How often are you in a violent situation? One where someone is hitting, kicking, stabbing or doing something else?	0.711

#### 2.3.3 Social media violence exposure

The initial scale for exposure to social media violence consisted of three questions. However, because the Cronbach’s Alpha was too low, we decided to use two of the items separately that directly measured exposure to violence on social media (Field, 2018). The first item was ‘How often in the last 12 months have you seen violent content on social media, like hitting, kicking, stabbing or something else?’. Answers ranged from 0 = never, 4 = more than 10 times. Thus this item measures “*violent social media exposure in general*,” and henceforth we will refer to it as such. The second item was ‘How many friends do you follow who use physical violence and post this on social media?’ Thus this item measures “*violent social media exposure by friends*” and henceforth we will refer to it as such. Answers ranged from 0 = none, 4 = more than 20. These two separate measures for online violence exposure were used in separate stepwise regression analyses (more details in the strategy of analysis).

### 2.4 Strategy of analysis

SPSS Version 27 was used to conduct all the analyses (IBM, Inc.). We mean-centered the peer deviancy variable since it would be used to compute the interaction term (Field, 2018). To interpret the regression coefficients, we used: 0.10: small effect; 0.30: medium effect; 0.50: large effect (Field, 2018).

#### 2.4.1 Stepwise (“blockwise”) regression

We had theoretical justification (DNERM & GAM) to prioritize certain predictors, and hence we selected a stepwise regression approach to be able to identify unique (relative) contributions of our predictors, while holding others constant. This technique allowed us to gauge incremental insight [i.e., extra variance (Δ*R*^2^)] that our key predictors add above and beyond other variables [e.g., gender or the main effects]. By entering our control variables in first block, we isolate the unique effect of our key predictors which were added in the 2nd block, and then subsequently adding the interaction terms in the 3rd block, we isolate the unique effects of these interaction terms. As customary, the criteria used for the model (“block”) comparisons (*within* each stepwise regression analysis), was the *R*-square difference (Δ*R*^2^) after each block was added. For example, we settled for the model without the interaction, if the Δ*R*^2^ was not significant in which the interaction was included (Field, 2018). We ran 4 of these stepwise regression models (models 1–4; see below), which was preceded by a main effects model (model 0).

#### 2.4.2 Model 0: main effect of violent social media exposure on real-life violence when controlling for gender

To test the hypotheses about the association between exposure to violence on social media and physical violence in real life, stepwise regression analyses were conducted to investigate this main effect while controlling for gender (Model 0). That is, the predictor variable exposure to social media violence and the control variable gender were included in a stepwise regression to predict the dependent variable real-life violence (i.e., main effect analysis). This main effect analysis was done twice, once for the independent variable social media violence in general and once for the variable social media violence by friends. Thereafter, the following 4 stepwise regression models were conducted to investigate the interaction effects.

#### 2.4.3 Model 1: violent social media exposure in general × developmental stage

In the first stepwise regression analysis (model 1), we first checked if exposure to violent social media content in general predicts the dependent variable physical violence in real life and if developmental stage moderates this link. Gender was included as a control variable in block one. In block two, the variables violent social media exposure in general and developmental stage were added. Lastly, the interaction term between exposure to violence on social media and developmental stage was added in block three.

#### 2.4.4 Model 2: violent social media exposure by friends × developmental stage

In the second stepwise regression analysis (model 2), the second independent variable (social media violence by friends) was used to predict the dependent variable physical violence in real life, and again we checked whether developmental stage moderates this link. Gender was treated as a control variable and therefore included in block 1. In block two, the predictor variables violent social media exposure by friends developmental stage were added. Finally, in block three, the interaction term between exposure to violence on social media and developmental stage (i.e., moderator variable) was added.

#### 2.4.5 Model 3: violent social media exposure in general × peer deviancy

In the third stepwise regression analysis (model 3), we checked if exposure to social media violence predicts the dependent variable physical violence in real life and if peer deviancy moderates this link. Gender was treated as a control variable again and was therefore included in block one. The variables exposure to social media in general and peer deviancy were added into the model in block two. Finally, in block three, the interaction term between exposure to violence on social media and peer deviancy was added.

#### 2.4.6 Model 4: violent social media exposure by friends × peer deviancy

In the final stepwise regression analysis (model 4), the independent variable social media violence by friends was used to predict the dependent variable physical violence in real life. Peer deviancy was included as a moderator and gender as a control variable. In block one gender (control variable) was included. In block two, the variables exposure to social media by friends and peer deviancy were added. Finally, in block three, the interaction term between exposure to violence on social media and peer deviancy (moderator variable) was added.

#### 2.4.7 Assumption checks

The assumptions for regression were checked (Agresti and Franklin, 2015). First histograms and scatterplots were used to check the assumptions for multiple stepwise regression (den Van Berg, n.d.). All the participants filled in the questionnaire individually, therefore their answers are considered independent. The assumption for normality was not met, but because of the Central Limit Theorem, this does not cause problems (*N* < 30) (Kothari, 2004). No pattern in the variance of the residuals could be seen, and thus the assumption for homoscedasticity was met. The assumption for linearity was met since no curve in the residuals could be observed. Finally, most of the variables did not correlate highly and/or had VIF values <10, so the final assumption for multicollinearity was met (Field, 2018).

## 3 Results

### 3.1 Descriptive statistics

The mean scores for the used variables were as follows: physical violence [M = 1.346 (SD = 0.653)], peer deviancy [M = 0.003 (SD = 0.772)], violent social media exposure from friends [M = 1.119 (SD = 0.417)], and violent social media exposure in general [M = 2.804 (SD = 1.523)]. A total of 67.7% of the participants was exposed to violent social media content at least once, while 26.7% “followed” friends on social media who post violent content themselves. All variables of interest were significantly correlated and in the expected direction (see Table 3 for the correlations).

**Table 3:** Correlations of variables.

	1	2	3	4	5
1. Gender	–				
2. Violence	−0.202**	–			
3. Peer deviancy	−0.145*	0.745**	–		
4. Social media violence in general	−0.040	0.237***	. 309**	–	
5. Social media violence by friends	−0.209**	0.430***	0.490**	0.225**	–

*p < 0.05, **p < 0.01, ***p < 0.001.

### 3.2 Model 0: Main effect of violent social media exposure on real-life violence when controlling for gender

#### 3.2.1 Violent social media exposure in general

A significant proportion of explained variance was found when adding social media violence in general and gender to the model (*R*^2^ = 0.093, *p* < 0.001; Table 4). These variables account for 9, 3% of variation in youth violence in real-life. The model described the data well *F*(2,229) = 11, 71, *p* < 0.001. Exposure to social media violence is a statistically significant predictor, with a small-medium effect size (sr^2^ social media = 0.228), gender has a small significant effect size (sr^2^ gender = −0.193).

**Table 4:** Summary of stepwise regression analysis of model 0: main effect of violent social media exposure in general on real-life violence when controlling for gender.

	*B*	*β*	*t*	sr^2^	R2	∆*F*
					0.093	11.709***
SM violence in general	0.097	0.228***	3.627	0.228		
Gender^a^	−0.272	−0.194**	−3.074	−0.193		

SM, social media.

a Male = 0, Female = 1, ∆*F* is change in *F*; sr^2^ is semi-partial correlation, sr^2^ = 0.10 (small effect), sr^2^ = 0.30 (medium effect), sr^2^ = 0.50 (large effect) (Field, 2018); *p < 0.05, **p < 0.01, ***p < 0.001.

#### 3.2.2 Violent social media exposure by friends

A statistically significant proportion of explained variance was found when adding exposure to social media violence by friends and gender to the model (*R*^2^ = 0.189, *p* < 0.001; Table 5). They account for 18.9% of the variation in youth violence. The model described the data well *F*(2,229) = 26.67, *p* < 0.001. Exposure to social media violence was a significant predictor with a medium effect size (sr^2^ social media = 0.385), while the control variable gender was not significant.

**Table 5:** Summary of stepwise regression analysis of model 0: main effect of violent social media exposure by friends on real-life violence when controlling for gender.

	*B*	*β*	*t*	sr^2^	R2	∆*F*
					0.189	26.666***
SM violence by friends	0.639	0.394***	6.470	0.385		
Gender^a^	−0.166	−0.118	−1.942	−0.116		

SM, social media.

a Male = 0, Female = 1, ∆*F* is change in *F*; sr^2^ is semi-partial correlation, sr^2^ = 0.10 (small effect), sr^2^ = 0.30 (medium effect), sr^2^ = 0.50 (large effect) (Field, 2018); *p < 0.05, **p < 0.01, ***p < 0.001.

### 3.3 Model 1: Violent social media exposure in general × developmental stage

Adding *gender* as a control variable in block 1 has a statistically significant impact on the model (*R*^2^ = 0.041, *p* = 0.002) and accounts for 4.1% of the variation in violence in youth. The model described the data well *F*(1,230) = 9.74, *p* = 0.002. The semi partial correlation for gender (sr^2^ = −0.202) indicates a significant small to medium effect (Field, 2018). Males are more likely to use physical violence.

In the second block, the variables exposure to *social media violence in general* and *developmental stage* (adolescents vs. emerging adults) were added. In this block a statistically significant proportion of explained variance was found (*R^2^* = 0.220, *p* < 0.001; Table 6), with an *R*^2^ change of 0.179. Adding these variables accounts for a 17.9% change in violence in youth [*F*change(2,228) = 26.15, *p* < 0.001]. Both exposure to violent social media content and developmental stage had a significant medium effect on youth violence (sr^2^ social media violence = 0.239; sr^2^ developmental stage = −0.356). Exposure to violent social media content in general predicts violence in real-life. Emerging adults (18–24-years-olds) show less physical violent behavior than adolescents (16–17-year-olds). The control variable gender does not have a significant effect in this second block.

**Table 6:** Summary of stepwise regression analysis of model 1: violent social media exposure in general × developmental stage.

	*B*	*β*	*t*	sr^2^	R2	∆*R*^2^	∆*F*
Block 1					0.041	0.041	9.742**
Gender^a^	−0.284	−0.202**	−3.121	−0.202			
Block 2					0.220	0.179	26.152***
Gender^a^	−0.070	−0.050	−0.784	−0.046			
Social media violence	0.102	0.239***	4.085	0.239			
Developmental stage	−0.500	−0.384***	−6.089	−0.356			
Block 3					0.246	0.026	7.968**
Gender^a^	−0.075	−0.053	−0.860	−0.050			
Social media violence	0.178	0.419***	4.877	0.281			
Developmental stage	−0.500	−0.384***	−6.183	−0.356			
SM violence * D stage	−0.139	−0.242**	−2.823	−0.163			

SM, social media; D, developmental.

a Male = 0, Female = 1, ∆*R*^2^ is change in *R*^2^, ∆*F* is change in *F*; sr^2^ is semi-partial correlation, sr^2^ = 0.10 (small effect), sr^2^ = 0.30 (medium effect), sr^2^ = 0.50 (large effect) (Field, 2018); *p < 0.05, **p < 0.01, ***p < 0.001.

For the third and last block, the interaction term between violent social media exposure in general and developmental stage was added to the model. This third block is a statistically significant predictor of violence in youth (*R^2^* = 0.246, *p* = 0.005). The *R*^2^ change value was 0.026, which indicates that adding the interaction term accounts for 2.6% of the change in youth violence. The interaction term had a significant contribution to youth violence [*F*change(1,227) = 7.97, *p* = 0.005]. In conclusion, the significant link between exposure to social media violence in general and youth violence, is stronger for adolescents (versus emerging adults). In other words, these results show that adolescents show more violent behavior in real life when exposed to social media violence in general than emerging adults.

### 3.4 Model 2: Violent social media exposure by friends × developmental stage

The first block when only *gender* is added as a control variable to account for levels of real-life youth violence, is the same as in model 1 in the previous paragraph, and thus has the same results. Namely, males use physical violence more than females.

In the second block, the predictor variables *violent social media exposure by friends* and *developmental stage* (adolescents vs. emerging adults) were added. In this block, a statistically significant proportion of explained variance was found (*R*^2^ = 0.276, *p* < 0.001; Table 7). The *R*^2^ change was 0.235. Adding these predictor variables accounted for a 23.5% change in violence in youth *F*change(2,228) = 37.06, *p* < 0.001. Both of these predictor variables had a significant medium effect on youth violence (sr^2^ social media violence = 0.337; sr^2^ developmental stage = −0.295). These results suggest that the more friends youth are exposed to that post violent social media content, the more violence youth would engage in in real-life. Emerging adults (18–24-years-old) showed less physical violent behavior than adolescents (16–17-year-old). Gender does not have a significant effect in this second block.

**Table 7:** Summary of stepwise regression analysis of model 2: violent social media exposure by friends × developmental stage.

	*B*	*β*	*t*	sr^2^	R2	*∆R^2^*	*∆F*
Block 1					0.041	0.041	9.742**
Gender^a^	−0.284	−0.202**	−3.121	−0.202			
Block 2					0.276	0.235	37.064***
Gender^a^	−0.010	−0.007	−0.121	−0.007			
Social media violence	0.565	0.349***	5.978	0.337			
Developmental stage	−0.418	−0.322***	−5.238	−0.295			
Block 3					0.280	0.004	1.320
Gender^a^	−0.020	−0.015	−0.236	−0.013			
Social media violence	0.526	0.324***	5.231	0.295			
Developmental stage	−0.392	−0.301***	−4.710	−0.265			
SM violence * D stage	0.346	0.073	1.149	0.065			

SM, social media; D, developmental.

a Male = 0, Female = 1, ∆*R*^2^ is change in *R*^2^, ∆*F* is change in *F*; sr^2^ is semi-partial correlation, sr^2^ = 0.10 (small effect), sr^2^ = 0.30 (medium effect), sr^2^ = 0.50 (large effect) (Field, 2018); *p < 0.05, **p < 0.01, ***p < 0.001.

In the third and final block, the *interaction term* between violence social media exposure by friends and developmental stage was added. This third block was not a statistically significant contributor to violence in youth (*β* = 0.073, *p* = 0.252). The *R*^2^ change value was 0.004, which indicates that adding the interaction term accounts for 0.4% of the change in youth violence, and it did not significantly contribute to youth violence *F*change(1,227) = 1.32, *p* = 0.252.

Considering the interaction effect in block 3 was not significant, we settled for the results of block 2 *without* the interaction effect. In sum, we found that both “violent social media exposure by friends” and “developmental stage” predict violence in real-life while controlling for gender.

### 3.5 Summary of models 1 and 2

In sum, the results of the two items for exposure to social media violence in general (model 1) or by friends (model 2) yield only partially similar results. That is, both of these violent social media exposure variables predict violence in real-life when effects of gender are accounted for. The strength of the link between exposure to social media violence *in general* and real-life violence is moderated by developmental stage (model 1). However, the strength of the effect of number of *friends* who post violent content is not moderated by developmental stage (see model 2). Specifically, model 1 demonstrated that adolescents (versus emerging adults) show more violent behavior in real life when exposed to social media violence in general, but developmental stage does not matter for the significant effect of violent social media exposure by friends on real-life violence (model 2).

### 3.6 Model 3: Violent social media exposure in general × peer deviancy

The first block of this model had the same results as mentioned above by model 1, when *gender* was added as a control variable. Namely, males use physical violence more than females.

In the second block, the variables *exposure to social media violence in general* and *peer deviancy* were added. A statistically significant proportion of explained variance was found (*R*^2^ = 0.560, *p* < 0.001; Table 8). An *R*^2^ change of 0.520 was calculated. Social media violence and peer deviancy account for a 52% change in violence in youth [*F*change(2,228) = 134.671, *p* < 0.001]. When inspecting the variables separately, exposure to violent social media did not show a statistically significant effect on youth violence (*β* = 0.009, *p* = 0.839), instead peer deviancy had a large significant effect on youth violence (sr^2^ = 0.684). This model shows that having deviant peers predict higher levels of physical violence in youth. Gender accounted for a small, but significant effect in violent behavior (sr^2^ = −0.106), suggesting that males are more violent than females.

**Table 8:** Summary of stepwise regression analysis of model 3: violent social media exposure in general × peer deviancy.

	*B*	*β*	*t*	sr^2^	R2	*∆R^2^*	*∆F*
Block 1					0.041	0.041	9.742**
Gender^a^	−0.284	−0.202**	−3.121	−0.202			
Block 2					0.560	0.520	134.671***
Gender^a^	−0.150	−0.107*	−0.2.410	−0.106			
Social media violence	0.004	0.009	0.204	0.009			
Peer deviancy	0.624	0.724***	15.567	0.684			
Block 3					0.560	0.000	0.078
Gender^a^	−0.152	−0.108*	−2.421	−0.107			
Social media violence	0.003	−0.008	0.166	0.007			
Peer deviancy	0.630	0.731***	13.659	0.601			
SM violence * peer D	−0.008	−0.014	−0.280	−0.012			

SM, social media; D, deviancy.

a Male = 0, Female = 1, ∆*R*^2^ is change in *R*^2^, ∆*F* is change in *F*; sr^2^ is semi-partial correlation, sr^2^ = 0.10 (small effect), sr^2^ = 0.30 (medium effect), sr^2^ = 0.50 (large effect) (Field, 2018); *p < 0.05 **p < 0.01, ***p < 0.001.

For the last block, the interaction term between social media exposure in general and peer deviancy was additionally included. This third block was not statistically significant (*R*^2^ = 0.560, *p* = 0.780). The interaction term did not change the violence in youth, a *R*^2^ of 0.000 was the result [*F*change(1,227) = 0.078, *p* = 0.780].

In sum, since the interaction effect was not significant, we opted for the results in block 2 *without* the interaction effect, in which we found that exposure to social media in general does not predict real-life violence when peer deviancy is also taken into account. That is, only peer deviancy (and gender) predicted violence in real life.

### 3.7 Model 4: Violent social media exposure by friends × peer deviancy

Once more, the results of the first block were the same as presented earlier when including the control variable *gender* first. That is being a male (versus female), predicted more youth violence.

In the second block the variables exposure to *social media violence by friends* and *peer deviancy* were added. In this block a statistically significant proportion of explained variance was found (*R^2^* = 0.563; Table 9) with a *R*^2^ change of 0.523. Adding these variables accounts for a 52.3% change in violence in youth [*F*change(2,228) = 136.393, *p* < 0.001]. When inspecting the variables separately, exposure to violent social media by friends did not show a statistically significant effect on youth violence (sr*^2^* = 0.056). Meanwhile, peer deviancy had a large significant effect on youth violence (sr*^2^* = 0.612). This block shows that having deviant peers, predicts higher levels of physical violence in youth. Gender accounted for a small, but significant effect in violent behavior (sr*^2^* = −0.095), showing that males are more violent than females.

**Table 9:** Summary of stepwise regression analysis of model 4: violent social media exposure by friends × peer deviancy.

	*B*	*β*	*t*	*sr* ^2^	R2	*∆R^2^*	*∆F*
Block 1					0.041	0.041	9.742**
Gender^a^	−0.284	−0.202**	−3.121	−0.202			
Block 2					0.563	0.523	136.393***
Gender^a^	−0.136	−0.097*	−0.2.163	−0.095			
Social media violence	0.105	0.064	1.273	0.056			
Peer deviancy	0.601	0.697***	13.978	0.612			
Block 3					0.568	0.004	2.274
Gender^a^	−0.137	−0.098*	−2.184	−0.095			
Social media violence	0.234	0.144	1.973	0.086			
Peer deviancy	0.614	0.712***	14.040	0.613			
SM violence * Peer D	−0.095	−0.110	−1.508	−0.066			

SM, social media; D, deviancy.

a Male = 0, Female = 1, ∆*R*^2^ is change in *R*^2^, ∆*F* is change in *F*; sr^2^ is semi-partial correlation, sr^2^ = 0.10 (small effect), sr^2^ = 0.30 (medium effect), sr^2^ = 0.50 (large effect) (Field, 2018); *p < 0.05, **p < 0.01, ***p < 0.001.

For the last block, the interaction term between social media exposure by friends and peer deviancy was additionally included. This third block was not statistically significant (*R*^2^ = 0.568, *p* = 0.133). The interaction term only accounted for a *R*^2^ change value of 0.004, which indicates that adding the interaction variable accounts for 0.4% of the change in youth violence [*F*change(1,227) = 2.274, *p* = 0.133]. In sum, and similar to model 3: peer deviancy was not a significant moderator, but it had a significant main effect on violence in real-life.

Considering the interaction effect in block 3 was not significant, we settled for the results of block 2 *without* the interaction effect. In sum, we found that while peer deviancy was not a significant moderator, it did have a significant main effect on violence in real-life. However, *exposure to social media violence by friends* did not have a significant main effect when it was simultaneously included it the same model with peer deviancy (see discussion section for related methodological considerations). Of note is the large correlation (*r* = 0.490) between peer deviancy and *exposure to social media violence by friends,* as this may (partially) explain why only one of these variables was significant when estimated in the same model. We elaborate on this possible caveat in the Discussion section.

### 3.8 Summary of models 3 and 4

For both models, regardless of who is posting the violent social media content: peer deviancy was not a significant moderator in the link between exposure to violent social media content and violence in real-life.

## 4 Discussion

Social media platforms are widely used, and especially youth are very active online. However, social media does not only have positive aspects. For example, youth can be exposed to violence via social media, which they might have otherwise not been exposed to. The question addressed in this paper is whether such violent social media exposure predicts real-life violence and whether this potential relation is especially prevalent for more vulnerable populations such as younger youth and youth who have deviant peers in real-life. Surprisingly this question has remained underexplored in quantitative publications on the influence of social media. Inspired by theoretical frameworks such as social learning theories, GAM and DNERM (Bandura, 1977; Anderson and Bushman, 2002; Defoe, 2021), we hypothesized that exposure to violent social media will be associated with more youth violence in real-life. We controlled for gender and used two separate measures of exposure to violent social media content: (1) violent social media exposure in general and (2) violent social media exposure by friends. Additionally, we examined whether developmental stage (adolescents aged 16–17-years vs. emerging adults aged 18–14-years) and peer deviancy served as moderators. Extrapolating from DNERM, we expect that social media influences (versus other media influences) would be most influential for real-world behavior (Defoe, 2021). We hypothesized that particularly youth would model the violence that they are exposed to online. We expected this finding because of the neuropsychological differences in adolescents and emerging adults. As stated by DNERM, the amount of self-control of an individual influences the regulation of risk-behavior (Defoe, 2021). Youth are expected to have less self-control (Defoe, 2021; Steinberg, 2010), therefore we expect them to use more real-life violence after exposure to violence on social media. Furthermore, we hypothesized that the association between exposure to social media violence and violence in real-life will be stronger for youth with more deviant peers, as demonstrated in research on traditional media (Fikkers, 2016). Finally, DNERM also suggests that “offline” social influences (e.g., peer deviance observed in the “real world”) would be more influential than (online) media influences for real-world behavior.

Stepwise regression analyses support the main hypothesis, as a significant association was found between the two measures of exposure to violent social media content and youth violence in real-life, even when controlling for gender. However, we only found partial support for the moderational hypotheses: while peer deviancy was not a significant moderator, developmental stage was a significant moderator but only when the “violent social media exposure in general” measure was used. Specifically, in support of DNERM (Defoe, 2021), the results showed that the link between violent social media exposure in general was stronger for adolescents (versus emerging adults). Further, the effects of social media exposure to violence became non-significant when controlling for peer deviancy—this result could be in part due to some (methodological) confounds that we address below. Below we also discuss theoretical implications of our findings regarding online influences when accounting for offline influences.

### 4.1 Exposure to social media violence and real-life violence

The findings support the hypothesis that exposure to violent social media content is related to more violence in real-life. This is in line with the link that was found between exposure to online risky behavior content (e.g., violence to others) and risky behavior in a real-life setting (Branley and Covey, 2017). These results raise the possibility that GAM, which addresses the relation between violent media *in general* and aggression, could be generalized to specifically violent *social* media exposure and violence. Namely, exposure to violent social media content could be seen as a situational input variable (stage 1), and therefore influence an individual’s internal state (affect/cognition/arousal) (stage 2), whom may respond in a violent or non-violent manner (stage 3) (see Anderson and Bushman, 2002; Allen et al., 2018). Or as DNERM posits, exposure to social media is an indirect form of exposure, that can be used as training ground for maladaptive risk behavior, such as violence (Defoe, 2021). DNERM further suggests a cue-reactivity hypothesis, in which indirect exposure to a cue of violence on social media may elicit the desire, normalization and acceptance of youth to try out such behaviors in real-life (Defoe, 2021). Importantly, our results suggest that the general finding that exposure to violent films, programs, and videogames makes youth more violent, may be generalized to the social media context (Konijn et al., 2007; Hollingdale and Greitemeyer, 2014; Hopf et al., 2008). Of note is that the effect of social media exposure to violence appeared to be stronger for such exposure that was specifically coming from friends compared to such exposure in general (that is exposure that could come from non-friends or anyone). This would fit the assumption of differential association theory, that the transmission of deviant ‘definitions’ predominantly takes place within the most intimate associations (Sutherland, 1947).

### 4.2 A moderating effect of developmental stage?

Interestingly, the two indicators of violent social media exposure produced different results when developmental stage was investigated as a moderator. That is, the strength of the link between exposure to social media violence *in general* and real life violence *is* moderated by developmental stage; while the strength of the effect of number of *friends* who post violent social media content on real-life violence is *not* moderated by developmental stage. Specifically, adolescents (versus emerging adults) show more violent behavior in real life when exposed to social media violence in general. Extrapolating from DNERM, this result emerged perhaps because adolescents are still developing their identity and are curious about the (online) world around them. And as a result, when exposed to violent content on their social media, adolescents may quicker try out that behavior in real-life compared to an (emerging) adult (Defoe, 2021). DNERM suggests that risk behavior such as engagement in violence, could be viewed by some as having a rewarding component, especially if it is observed in one’s environment. That is, violence in one’s environment can imply that their social circle endorses and accepts violence, and thus engaging in violence could be rewarding because it may make an individual more accepted within their social circle. Hence, especially for youth [versus (emerging) adults] who have a need for exploration (e.g., due to their ongoing identity formation; Defoe, 2021), might decide to experiment with violence they are exposed to in their environment to learn through their own experience whether engaging in violence is beneficial for them. And they may choose to use this experience to ultimately decide whether they fit within their (violent) environment (e.g., Erikson, 1963; Defoe, 2021; Defoe et al., 2015).

However, a caveat is that we found that developmental stage does not matter for the significant effect of violent social media exposure *by friends* on real-life violence. These results suggest that on the one hand, if your friends are posting violent content on social media, then being exposed to this would impact youths’ own violence regardless of the developmental stage of the youth. On the other hand, if the violent social media content that youth are exposed to is posted not specifically by their friends perse, then it would particularly be younger youth (i.e., adolescents) who would model this violent behavior in real-life. All in all, these results suggest that when it comes to the effect of violent social media exposure on real-life violence, it may matter who (friends versus anyone) is posting the violent content, but it also matters who the audience (e.g., adolescents versus [emerging] adults) is.

The current study is of added value because it is apparently the first to investigate the impact of developmental stage on the strength of the association between exposure to social media violence and violence in youth. For this reason, it was also not possible to compare our results to empirical studies addressing the same link, since these studies do not exist. Nevertheless, our significant moderational results of developmental stage are supported by DNERM (Defoe, 2021).

### 4.3 A moderating effect of peer deviancy?

The current findings suggest that peer deviancy does not moderate a main effect between exposure to social media violence and violence in real-life. Thus the findings do not confirm our hypothesis that the association will be stronger for youth with more deviant peers than for youth with fewer deviant peers, which was inspired by the findings in Fikkers (2016), which also specifically investigated violence but focused on traditional media (e.g., TV) and games. Based on the current findings that did not find a significant effect of peer deviancy, it cannot be concluded as yet whether the findings of Fikkers (2016) can be generalized to social media, more specifically. To what extent such a generalization is probable is important to establish considering that social media is a different more “intimate” and “interactive” type of media (compared to traditional media), and which thus presumably would have different effects on real-world behavior.

Finally, despite not finding an interaction effect, it is important to note that violence in real-life was predicted by peer deviancy, a finding that is not surprising considering prior research on deviant peer influences in “real-life” (Warr, 2002; Hawkins et al., 1998; Albert and Steinberg, 2011; Defoe, 2021). Our results further show that when peer deviancy is accounted for, the effect of exposure to violent social media content on real-life violence becomes non-significant. This result can be extrapolated from DNERM that posits that real-world influences would be larger than online influences (Defoe, 2021), but it is not consistent with a similar cross-sectional survey study that found a (main effect) link between exposure to online violence to others and violence in real-life among *(emerging) adults*, even while controlling for peer deviancy (Branley and Covey, 2017). In any case, besides for the substantially older sample used in Branley and Covey (2017) compared to that sample in our study, one possible methodological explanation for why we no longer found a significant effect of violent social media exposure when accounting for peer deviancy could be attributed to “all of the violent social media content that youths are exposed coming from their deviant friends.” In that case, it could be the deviancy of friends that matters most and not what they post on social media. Another possibility could be that having deviant friends already exert the most fundamental peer influence on individual physical violence, rendering the effects of social media exposure redundant. If these explanations are right, then interventions for youth violence should better focus on the influence of deviant peers in real-life, then on (or also on) exposure to violence exposure online (via social media).

However, of note is that the dominating effect of peer deviancy can also be explained in alternative ways. One alternative explanation is that deviant friendships are also formed by “peer selection.” Individuals search for friends who are like themselves, including delinquent behavior (see, e.g., Gallupe et al., 2019; Ragan, 2019). This would imply that deviancy of friends are not only a cause of individual deviant behavior (in this case physical violence), but also a consequence. Since this study is cross-sectional, it does not rule out that such reversed effects may also exist. Further, it should be kept in mind that individuals who are involved in violent behavior can also be the deviant peers of other individuals in the sample, which would further flaw the association between deviancy of peers and violence. Thirdly, a considerable portion of violence takes place in groups or with other individuals (see, e.g., Conway and McCord, 2002; Van Mastrigt and Carrington, 2019), and it has been pointed out that this is to be expected especially for youth, simply because they spend a lot of time with each other (Defoe, 2016). Considering this, being violent automatically implies that one (particularly youth) has peers who are also violent, but this does not mean that this association can be interpreted as causal.

And finally, future studies could also consider the finding of peer deviancy overpowering the effects of violent social media content exposure (by friends) in context of our developmental stage moderation finding that show that *particularly adolescents’ (versus emerging adults) engagement real-life violence is impacted by violent social media content*. Then the burning unanswered question that requires follow-up research is whether this developmental stage moderation finding would remain even when peer deviancy is accounted for. Future studies with a larger sample size could investigate this 3-way-interaction hypothesis (i.e., violent social media content exposure × peer deviancy × developmental stage). This hypothesis would also be a more complete test of DNERM, which postulates effects of online exposure to risk behaviors (such as violence) on real-life risk behaviors, but particularly for younger youth versus older youth (and potentially particularly for youth with higher levels of impulsivity) (Defoe, 2021). All in all, considering these alternative explanations, it is not yet possible to draw definitive conclusions about the actual effects of social media exposure on violence in combination with the effects of peer deviancy (in real-life).

### 4.4 Strengths, limitations and future research directions

This is one of the first studies that focused on the implications that exposure to violence on *social* media could have on youth violence in real-life, and apparently the first quantitative study among the relevant qualitative studies we located. While aiming to fill this research gap, the current study further focused on a unique combination of constructs. Namely, exposure to social media violence (in general and by friends), developmental stage (adolescents versus emerging adults), peer deviancy and youth violence in real-life. Specifically, this study not only investigated a potential link from exposure to violent social media content and youth real-life violence, but it is also the first to consider moderating effects of developmental stage and peer deviancy in this understudied link.

There are various limitations in the current study that need to be considered. One limitation is that our measure of violence is limited to physical acts of violence and aggression focused on physical force. Our findings do not necessarily extend to other forms of violence, including sexual offenses or harassments, bullying and so on. Future research is needed to see whether the findings of our study can be extended to violence in a broader sense.

Another limitation is the new measure used for violent social media content exposure (since such scales appear to be non-existent). A single item was used for both indicators of this construct, while in reality, exposure to violence may come in different forms, on different platforms and with different frequencies. We opted not to create a scale consisting of multiple items since the Cronbach’s alpha turned out to be very low. Conclusions can be less reliable when drawn based on one item (Agresti and Franklin, 2015). Nevertheless, numerous studies show that single-item scales lead to similar results as scales with more items (Brailovskaia and Margraf, 2020). Still it is clear that (more) multiple-item scales of violent social media content exposure need to be developed in the future, since we could not locate any.

Another possible limitation in hindsight is that although not intended, the way participants responded to our measure of peer deviancy (example item: “*How many times in the last 12 months did one of your friends use physical violence, like hitting, kicking, stabbing or something else?”*) might already be confounded with peer deviancy online (e.g., via social media), making our separate measure for violent social media (by friends) redundant. And hence leading to its non-significant effects when accounting for our overarching peer deviancy variable as a control. Obviously, youths are also likely exposed in real-life to the violence of those friends who are posting such violent content on social media. This is an important caveat that future studies on online influences could consider. That is, we would like to caution future studies that aim to investigate a potential additive or interacting effect of peer deviancy on the influence of exposure to online deviancy (e.g., violence) that their peer deviancy measure should explicitly exclude any type of online peer deviancy.

This study solely relies on self-reported data, this can also be viewed as a limitation to this study. Part of the participants could have answered the questionnaire with what they thought would reflect positively on them or because they feared that it could become known to other people. They could have forgotten events or think it was further in the past. However, although self-report measures are not perfectly reliable, they are generally seen as valid and more reliable than official records (Gomes, 2025).

In closing, limitations regarding our statistical approach that need noting is that although stepwise regression was an appropriate method for our aims, its generated results can be unreliable in some cases. For example, this may be the case if (a) the change in *R*-Square is large or significant while the added predictors are not statistically (and/or theoretically) significant, or (b) if including too many models (i.e., “blocks”) leads to model comparison complexities. But we did not observe such *R*-Square change issues or experienced multiple comparison issues. We circumvented the latter by using limited blocks. And finally, no causal inferences can be drawn from the results of the current study, considering that it is not an experiment (Maruyama and Ryan, 2014). Hence, future research could explore whether there are ethical ways to investigate potential causal links between social media violence exposure and youth violence in real-life. At minimum, longitudinal research that can disentangle direction of effects also ought to be a next step in this type of research, as it can help confirm some of the alternative explanations we raised earlier.

## 5 Conclusion

Considered together, the current study demonstrated that exposure to violence on social media may predict youth violence in real-life. The results also demonstrated that when it comes to the effect of violent social media exposure on real-life violence, it may matter who (friends versus non-friends/anyone) is posting the violent content, but it also matters who the audience [e.g., adolescents versus (emerging) adults] is, and whether peer deviancy in general is controlled for.

Specifically, we found that while peer deviancy was not a significant moderator, developmental stage was a significant moderator—but not when the violent social media content was posted by friends of the youth. Namely, on the one hand, the association between violent social media exposure in general and engagement in violence in real-life is most relevant for adolescents (versus emerging adults). On the other hand, when violent social media content is posted *by friends*, exposure to this content was a predictor of real-life youth violence *regardless* of the developmental stage (i.e., it was a significant predictor for both adolescents and emerging adults). This is perhaps the case because youth are also exposed in *real-life* to the violence of those friends who are posting such violent content on social media. And if that is the case, then it is not surprising that when peer deviancy is accounted for, the association between exposure to violent social media content and youth violence in real-life becomes negligible. However, it is important that future research builds on the findings of the current study, as it may still be likely that even when controlling for peer deviancy, this link from “online to offline violence” may emerge particularly for adolescents considering that adolescents (versus emerging adults) were found to be more susceptible to violent social media content in general—albeit that the effect of real-world may be stronger (see DNERM; Defoe, 2021). This importance of a possible moderating role of developmental stage (and of impulsivity) is also emphasized DNERM (Defoe, 2021).

Taking into account the popularity of social media, the young age at which individuals begin using social media, and the easy access to violent content via such means, more research focusing on the relation between exposure to social media violence and youth violence in real-life is necessary. Particularly, longitudinal research is needed as a next step, since it may very well be the case that the direction of the link could be reversed. That is, engaging in violence in real-life may subsequently predict that youth will be exposed to more violence on social media. For example, youth who engage in violence may be more likely to search for such content on social media, and the algorithms in return may expose them even more to such content. Another example is that these youth who engage in violence are more likely to follow others (e.g., friends) on social media that similarly engage in violence and post such content on social media, which these youth subsequently become exposed to. Indeed bi-directional links between violent social media exposure on the one hand and engagement in violence in real-life on the other hand, can equally exist. Thus there remains a lot of scenarios to be investigated in the link we found between violent social media content exposure and engagement in violence in real-life. Considering that this study is the first to investigate this link, it contributed to providing some new insights—although many intriguing above-mentioned questions for future research were also raised while doing so.

## Data Availability

The raw data supporting the conclusions of this article will be made available by the authors, without undue reservation.
